# RETRACTED: Uner et al. Assessing the In Vitro and In Vivo Performance of L-Carnitine-Loaded Nanoparticles in Combating Obesity. *Molecules* 2023, *28*, 7115

**DOI:** 10.3390/molecules31111962

**Published:** 2026-06-05

**Authors:** Burcu Uner, Ahmet Dogan Ergin, Irfan Aamer Ansari, Melahat Sedanur Macit-Celebi, Siddique Akber Ansari, Hamad M. Al Kahtani

**Affiliations:** 1 Department of Administrative and Pharmaceutical Sciences, University of Health Science and Pharmacy in St. Louis, St. Louis, MO 63110, USA; 2 Department of Pharmaceutical Technology, Faculty of Pharmacy, Trakya University, 22030 Edirne, Turkey; 3 Department of Neuroscience, University of Turin, 10124 Turin, Italy; 4 Department of Pharmaceutical Nanotechnology, Institute of Health Sciences, Trakya University, 22030 Edirne, Turkey; 5 Department of Drug Science and Technology, University of Turin, 10124 Turin, Italy; irfanaamer.ansari@unito.it; 6 Department of Nutrition and Dietetics, Faculty of Health Sciences, Ondokuz Mayıs University, 55270 Samsun, Turkey; sedanurmacit@gmail.com; 7 Department of Pharmaceutical Chemistry, College of Pharmacy, King Saud University, Riyadh 11451, Saudi Arabia; sansari@ksu.edu.sa (S.A.A.); ahamad@ksu.edu.sa (H.M.A.K.)

The journal retracts the article titled “Assessing the In Vitro and In Vivo Performance of L-Carnitine-Loaded Nanoparticles in Combating Obesity.” [1], cited above.

Following publication, concerns were brought to the attention of the Editorial Office regarding image duplication and the accuracy and validity of the Institutional Review Board Statement published in the article [1].

Adhering to our standard procedure, the Editorial Office and Editorial Board conducted an investigation that confirmed inappropriate editing and partial duplication between figures presented in this article [1] and earlier publications [2,3], produced by a different research group. In addition, the Institutional Review Board of the University of Health Science and Pharmacy in St. Louis investigated the matter and concluded that the approval number in the study [1] was not valid. As a result, the Editorial Board have decided to retract this publication [1], as per MDPI’s retraction policy.

This retraction was approved by the Editor-in-Chief of *Molecules*.

Dr. Sedanur Macit-Çelebi, Dr. Hamad M. Alkahtani, Dr. Irfan Aamer Ansari, and Dr. Burcu Uner disagree with the retraction. Dr. Ahmet Dogan Ergin agrees with the retraction. The remaining author has not provided a comment. Individual contributions to the retracted work are detailed in the published article [1].
